# Invasive Salmonellosis by the Very Rare *Salmonella choleraesuis* in a Returning Traveler on a Tumor Necrosis Factor-**α** Inhibitor

**DOI:** 10.1155/2014/934657

**Published:** 2014-02-23

**Authors:** Uzoamaka A. Eke, Harry Conte, Paula Anderson, Robert W. Lyons

**Affiliations:** ^1^Division of Infectious Diseases, Washington University in St. Louis School of Medicine, St. Louis, MO 63110, USA; ^2^Department of Infectious Diseases, St. Francis Hospital, Hartford, CT 06105, USA; ^3^Department of Microbiology, St. Francis Hospital, Hartford, CT 06105, USA

## Abstract

*Salmonella choleraesuis* is one of the least commonly reported nontyphoidal salmonellae in the United States, accounting for only 0.08% and ranking lower than 20th place among all human source salmonellosis reported to the CDC in 2009. In the state of Connecticut, only 12 cases have been reported since 1998 and our case is the only case since 2008. We report a case of invasive Salmonellosis caused by *Salmonella choleraesuis* in a patient on an antitumor necrosis factor-**α** agent (adalimumab) who recently returned from a trip to the Dominican Republic.

## 1. The Patient

A 52-year-old white male with a history of Crohn's disease, nonalcoholic steatohepatitis, and liver cirrhosis, living in Hartford, CT, USA, presented in April 2011 with a four-day history of fever, chills, and severe headache all of which began approximately one week after his return from the Dominican Republic where he had travelled for a 10-day vacation with a group of family and friends. He had no abdominal pain or diarrhea but had some nausea and vomiting. He stayed at a “5-star resort” from where he obtained all his food. He drank only bottled water although he used ice which he obtained locally. He did not receive any vaccinations or prophylaxis prior to his travel. He had been on adalimumab (humira), 40 mg injections weekly, and 6-mercaptopurine, 75 mg by mouth daily for the past 2 years for crohn's disease diagnosed more than 20 years ago.

Physical examination was remarkable for a maximum temperature of 103.8. He had a normal abdominal examination. Pertinent laboratory findings include a WBC of 3,500/*μ*L and two sets of blood cultures that grew *Salmonella choleraesuis* which was susceptible to ceftriaxone and ciprofloxacin with a ciprofloxacin MIC reported as <0.2 *μ*g/mL. However nalidixic acid sensitivity was not reported. Stool culture had no growth. He had a normal CSF examination and a negative HIV ELISA.

The patient was initially treated with intravenous ceftriaxone with good clinical response, defervescing within 36 hours with no growth from blood cultures obtained two days after antibiotics were instituted. Since the organism was also susceptible to ciprofloxacin, the patient was discharged home on a two-week course of oral ciprofloxacin. However he returned to the hospital a month later with similar symptoms as well as recurrent bacteremia with the same organism. This time nalidixic acid sensitivity was reported as resistant so ciprofloxacin was not recommended for therapy. Further workup to evaluate for involvement of extra intestinal sites including a transesophageal echocardiogram and CAT scan of the chest and abdomen with IV contrast was unremarkable. His adalimumab was temporarily discontinued and he was discharged home on a one-month course of intravenous ceftriaxone and has done well without any recurrence after six months of follow up.

## 2. Literature Review and Discussion

**Figure 1:**
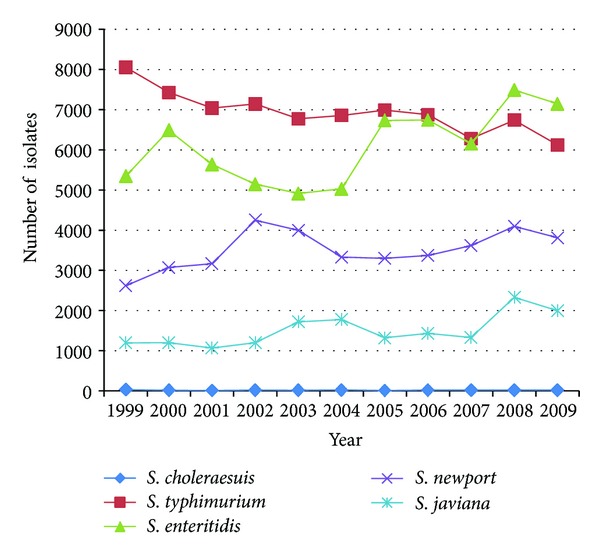
Laboratory-confirmed *Salmonella* isolates from human sources reported to CDC by serotype and year, 1999–2009; comparing the number of *S. choleraesuis* isolates to the most frequently reported serotypes.

*Salmonella choleraesuis* is a serotype of *Salmonella enterica sub specie enterica. S. choleraesuis* human infection is extremely rare in the United States. From the CDC public health laboratory information system (PHLIS) 2009 data, it accounted for only 0.08%, ranking lower than 20th place among all human salmonellosis isolates in the United States [1] (see Figure 1). Connecticut has only reported 12 cases since 1998 and our case is the only case since 2008 according to the Connecticut department of public health.

*S. choleraesuis* primarily causes enterocolitis and septicemia in pigs and is only very rarely isolated from other reservoirs; hence, most human infection is acquired from pigs [2, 3]. Studies have shown that human isolates of this organism are genetically and epidemiologically closely related to the isolates obtained from infected swine [2, 4]. The organism is shed by carrier and infected animals which can result in continued environmental contamination and reinfection of new animals by horizontal transmission [2, 3]. Studies citing US data from the beginning of the last decade report that *S. choleraesuis * was responsible for more than 90% of salmonellosis in swine [2, 3]. However, more recent CDC data from the 2009 annual *Salmonella* summary revealed that it caused only 2.4% of clinical porcine salmonellosis with *S. typhimurium var. 5-* being the highest reported at 26.6% [1].

Most case reviews on this organism are from Taiwan where it ranked as the second commonest *Salmonella* causing human disease for most of the 1990s with prevalence as high as 8% [2]. This trend continued until it began to decline in 2004 and thereafter, with a prevalence of 4.3% in 2004 and 0.84% in 2007 [5]. The decline has been attributed to the institution of strict antibiotic regulation in live stock by the Taiwan department of Agriculture in 2002 as well as the introduction of vaccination of pigs in 2004 with the live attenuated *S. choleraesuis* vaccine, suisaloral (Impfstoff-werk Dessau-Tornau GmbH, Germany) [4].

These reviews have shown that *S. choleraesuis* has a higher predilection than other *Salmonella* serotypes to cause primary bacteremia and focal invasive disease including endovascular infection especially in immunosuppressed patients with little or no intestinal manifestations as well as a tendency for recurrent infection [2, 6]. There has been no recent review of *S. choleraesuis* infection in the United States since the series published by Allison et al. from the Medical college of Virginia in 1969 which reviewed 19 cases diagnosed over 13 years where all the cases had bacteremia and all had negative stool cultures [6].

This case report describes the somewhat unusual clinical presentation of *Salmonella choleraesuis* when compared to other nontyphoidal salmonellosis. Whereas most other nontyphoidal salmonellae cause self-limited gastroenteritis with only 5% of patients developing bacteremia, *S. choleraesuis* causes primary bacteremia in up to 70% of patients and focal invasive, extra intestinal disease in 25–50% of patients with a paucity of gastrointestinal symptoms especially in the immunosuppressed, older patient [7, 8]. Multiple case reviews have shown that immunosuppression is the most significant risk factor for invasive salmonellosis due to this organism [7, 8]. There is an increasing report of invasive salmonellosis cases among patients on anti-TNF-*α* agents [9, 10]. Other risk factors include age above 50, liver cirrhosis, inflammatory bowel disease, systemic lupus erythematosus, and diabetes mellitus. Mycotic aneurysms, osteomyelitis, and pleuropulmonary infections are the commonest focal infections reported [6–8].

Recurrence after appropriate and adequate antimicrobial treatment is a feared clinical scenario in patients with *S. choleraesuis* infection [7] (see Table 1). Some of the risk factors associated with recurrence include preexisting tissue damage such as atherosclerotic plaques and diseased bones or joints. Timely surgical intervention in addition to antibiotics may prevent recurrence in patients with mycotic aneurysms and osteomyelitis [7].

In the United States, severe *Salmonella* infections including typhoid fever are treated with fluoroquinolones such as ciprofloxacin and third-generation cephalosporins such as ceftriaxone [1]. Resistance to nalidixic acid, an elementary quinolone, in enterobacteriaceae correlates with decreased susceptibility to ciprofloxacin (MIC ≥ 0.12 *μ*g/mL) and possible fluoroquinolone treatment failure. The resistance of nontyphi salmonellae to nalidixic acid and ceftriaxone in the US averages about 2% and 2.8%, respectively, whereas resistance to ciprofloxacin remains rare. On the contrary, *S. typhi* resistance to nalidixic acid in the US is remarkably higher at 69.1% [1]. There is no data on *S. choleraesuis* resistance to these antibiotics in the US probably because of the rarity of its occurrence here.

In Taiwan however, where *S. choleraesuis* is a major public health problem, studies have shown a rapidly increasing resistance to ciprofloxacin approaching 100% [4, 11]. A prospective, laboratory-based surveillance study of fluoroquinolone resistance in *S. choleraesuis* isolated from humans in four major teaching hospitals across Taiwan revealed an overall significant increase in resistance to ciprofloxacin from 32.3% in 2000 to 56.5% in 2001, 61.8% in 2002, and 71.8% in 2003 [11]. A case series of 47 patients with *S. choleraesuis* infection from a large university hospital in Taiwan revealed 100% resistance to Nalidixic acid, 55% resistance to ciprofloxacin, <5% resistance to ceftriaxone, but no resistance to cefepime, aztreonam, and the carbapenems [8]. This high rate of resistance to the quinolones is attributed in part to the use of quinolones in swine feed in Taiwan. These data therefore imply that extended spectrum cephalosporins remain the most reliable option for treating *S. choleraesuis* infection in these locations but this may not apply to the USA where ciprofloxacin resistance in nontyphi salmonellae remains rare [1].

Our patient may have acquired his infection from the Dominican Republic but the literature has not shown an association of this infection with this location besides one case report of a 70-year-old male who presented with endocarditis caused by *S. choleraesuis* after returning from the Dominican Republic [12]. In fact, there is no data on the occurrence of *S. choleraesuis* in pigs or humans in the Dominican Republic. Our patient did not report any ingestion of Pork or contact with swine during his travel. The ice he used during his travel may have been contaminated and may have been the source of his infection. Unfortunately, data on the travel history of the cases reported in Connecticut and other states of the United States are not available so it is unclear if these infections were acquired locally or abroad.

The index patient had multiple immunosuppressing conditions besides being on a biologic agent that increased his risk for invasive disease. He may likely have benefited from a pretravel evaluation for necessary vaccinations prior to his travel including the *Salmonella typhi* vaccine which he is also at a higher risk of developing invasive disease from given his immune status.

Nalidixic acid susceptibility testing was not done during the patient's initial presentation, the result of which may have precluded the use of ciprofloxacin in the first place. However it is not clear if the recurrence of his infection was a result of treatment failure due to a reduced susceptibility to ciprofloxacin or if it was a question of his immune status or if it was a case requiring a longer duration of treatment than the two weeks he initially received.

The 2011 CLSI guidelines did recommend nalidixic acid susceptibility testing as a form of screening in order to detect reduced susceptibility to ciprofloxacin in extraintestinal *Salmonella* species that are reported as susceptible to ciprofloxacin [13]. The problem however is that resistance to nalidixic acid does not always infer clinical failure with ciprofloxacin and nalidixic acid may not identify all the mechanisms of fluoroquinolone resistance [13, 14]. Hence in the 2012 CLSI guidelines, the need for nalidixic acid susceptibility testing was eliminated entirely by lowering the ciprofloxacin breakpoint (<0.06 *μ*g/mL) for *S. typhi* and extraintestinal *Salmonella* infections [13, 15].

It is possible that our patient may have benefited from a longer duration of antibiotic treatment than the two weeks he received during his initial presentation given his multiple immunosuppressing conditions. The important thing to remember however is that recurrent infection with this organism has not been associated with inadequate or inappropriate antibiotic therapy although it remains to be determined what length of antibiotic therapy is deemed adequate. Based on the data presented in this report, it appears prudent that a third or higher generation cephalosporin should be used to treat *S. choleraesuis* infection until the susceptibility of the organism to ciprofloxacin is determined if the use of the latter is being considered.

## 3. Conclusion

*S. enterica serotype choleraesuis* human infection is rare in the United States. It causes invasive disease especially in elderly, immunosuppressed patients, may not manifest with gastroenteritis, and has a high tendency for recurrence. There is still a lot that remains to be learned about the epidemiology, treatment, and prevention of recurrence of this infection especially in the United States and a travel history may be helpful to determine the source of infection. There is a need to address the travel requirements and evaluation of patients on anti-TNF-*α* and other biologic agents travelling to disease endemic locations.

## Figures and Tables

**Table 1:** Summary of clinical features of patients with recurrent *S. choleraesuis* infections. [7] Copyright © 2006 Cambridge University Press. Reprinted with the permission of Cambridge University Press.

Patient number	Age/sex	Underlying medical condition	Initial diagnosis	Disease recurrence (source of positive culture)	Interval between first presentation and second presentation
1	26/M	Bilateral femoral head avascular necrosis s/p total hip replacement	Osteomyelitis	Osteomyelitis (drain wound)	4 months
2	69/M	Diabetes mellitus, hepatocellular CA	Primary *S. choleraesuis* bacteremia	Mycotic aneurysm (blood, tissue)	3 years
3	71/M	Hypertension, bladder transitional cell CA, ESRD undergoing hemodialysis	Mycotic aneurysm	Mycotic aneurysm (blood)	2 months
4	50/M	Hypertension, aortic regurgitation s/p mechanical valve	Mycotic aneurysm	Mycotic aneurysm (blood)	6 months
5	48/F	Nil	Osteomyelitis	Osteomyelitis, enterocolitis (blood, stool)	5 months
6	65/M	Hepatitis B virus infection-related liver cirrhosis	Primary *S. choleraesuis* bacteremia	Spontaneous bacteria peritonitis (blood, ascites)	6 months
